# Differential Expression of Claudin 1 and 4 in Basal Cell Carcinoma of the Skin

**DOI:** 10.1155/2023/9936551

**Published:** 2023-01-20

**Authors:** T. Nakazawa, A. Hasegawa, T. Nagasaka, K. Yoshida, F. Guo, D. Wu, K. Hiroshima, M. Takeuchi

**Affiliations:** ^1^Departments of Pathology, Tokyo Women's Medical University Yachiyo Medical Center (TYMC), Yachiyo, Chiba 276-8524, Japan; ^2^Plastic Surgery, Tokyo Women's Medical University Yachiyo Medical Center (TYMC), Yachiyo, Chiba 276-8524, Japan

## Abstract

Basal cell carcinoma (BCC) is the most common human malignancy. The biological behavior of this entity is remarkably indolent. Claudin plays an important role in tight junctions, regulating paracellular passage of variable substance including growth factors and maintaining the polarity of epithelia. Up- or downregulated claudin expression has been reported in many cancers. Nevertheless, claudin expression in BCC of the skin remains unclear. We therefore examined the status of claudin 1 and 4 expressions in BCC and adjacent normal skin by immunohistochemistry (IHC). Our IHC results demonstrated high claudin 1 expression and low claudin 4 expression in 33 of 34 lower-grade BCCs. In lower-grade BCC, claudin 1 was increased and claudin 4 was decreased compared with the normal skin. Claudin 1 was inclined to be highly expressed in the membrane and cytoplasm of tumour cells in the periphery of tumour nest. Conversely, almost all lower-grade BCCs (33/34) and one of two higher-grade BCC lacked or showed focal positivity for claudin 4. These results imply that the expression pattern is characteristics of lower-risk BCC. Interestingly, one of the two higher-grade BCCs demonstrated the converse expression patterns of claudins, with decreased claudin 1 and increased claudin 4. The combination of immunohistochemical claudin 1 and 4 expression may offer a useful ancillary tool for the pathological diagnosis of BCC. Furthermore, membranous and intracellular claudins may present future therapeutic targets for uncontrollable BCC.

## 1. Introduction

Basal cell carcinoma (BCC) of the skin is the most frequent human cancers [1]. The prognosis of patients with BCC is exceedingly favorable. Neither recurrence nor metastasis usually occurs, while local recurrence occasionally occurs as a consequence of incomplete resection. BCC can show a wide variety of macro- and microscopic appearances. The World Health Organization (WHO) classification defines several BCC variants based on these morphological characteristics [1]. According to the risk of recurrence, these variants are largely divided into higher- and lower-risk categories. Lower-risk BCCs includes nodular, superficial, pigmented, infundibulocystic, and pigmented types. Higher-risk BCCs encompass basosquamous carcinoma, BCC with sarcomatoid differentiation and sclerosing/morphoeic, infiltrating, and micronodular types.

Tight junctions (TJs) are located in the cytomembrane and maintain cell-to-cell adhesion in epithelial and endothelial cells [2, 3]. TJs play an important role in proliferation, survival, apoptosis, and differentiation [4]. Physiologically, TJ proteins possess two major functions: “barrier” and “fence.” In more detail, the “barrier” function regulates the passage of ions, water, macromolecules, and cancer cells from the paracellular space. Dysregulation can contribute to edema, jaundice, diarrhea, and cancer metastasis. The “fence” function maintains an appropriate arrangement and compartmentalization between individual cells. Impairment of this function results in disruption of cell adhesion, arrangement, and polarity. Cancer cell readily metastasize under such conditions.

TJs are mainly composed of occludin, junctional adhesion molecules (JAMs), and claudin [5]. Claudins structurally include four transmembrane domains with two extracellular loops and N- and C-terminal regions in the cytoplasm [4]. The second extracellular loop contains a *Clostridium perfringens* endotoxin (CPE)-binding site. The PDZ (PSD-95/DLG/Zo-1) domain is located at the C-terminus and binds to scaffold proteins such as ZO-1, participating in protein-protein interactions and signal transduction. Twenty-seven variants of claudin have been identified to date. In particular, functions of claudin 1 are widely known to be critical in the skin and loss of this protein is lethal. Claudin 1-deficient transgenic mice died within 1 day after birth from dehydration [6]. Several previous studies have reported that normal epidermis weakly expresses claudin 4 in the stratum granulosum [7, 8]. However, the function of claudin 4 in normal skin remains under debate.

Little has been determined about the roles of claudins in skin tumours. Most reports regarding the claudin family have documented claudin 1 in cutaneous squamous cell carcinoma (cSCC) [7–10]. In particular, the physiopathological significance of claudin 4 is largely unclear because of a paucity of related articles. One report described spiradenoma as showing a distinctive immunostaining pattern [11]. Sato et al.suggested that claudin 4 possibly regulates keratinization in basaloid SCC and the SCC of oral lesions [12]. However, a review of the literature failed to identify reports describing the expression of claudin family members in BCC that purely arise from the skin. We therefore decided to study claudin 1 and 4 to clarify the state of expression in BCC in comparison with adjacent normal skin using immunohistochemistry (IHC) in paraffin-embedded formalin-fixed tissues.

## 2. Materials and Methods

### 2.1. Patients and Human Skin Tissues

We examined a total of 36 surgically resected specimens for skin tumour. The pathological diagnosis of BCC was made and variants were classified based on histopathological features in accordance with the World Health Organization Classification of Skin Tumours [1]. Variants were divided into lower grade (nodular, superficial, infundibulocystic, or fibroepithelial) or higher grade (infiltrating or micronodular). The age, sex, and prognosis of patients were obtained from the clinical data of Tokyo Women's Medical University Yachiyo Medical Center (TYMC). All skin samples were obtained from patients who underwent surgery at TYMC from January 2016 to December 2022.

All excised materials and biopsies were routinely processed and embedded in paraffin blocks. Sections were cut 4 µm thick and then stained with hematoxylin and eosin (HE) for routine pathological diagnosis. After microscopic observation, we selected paraffin blocks that included the maximum cut surface of BCCs for IHC. All samples were evaluated independently by experienced pathologists (TK and KH).

### 2.2. Immunohistochemistry

Four-micrometer-thick sections were cut from the paraffin blocks, deparaffinized, then pretreated with EDTA-buffer at pH 9.0 for 20 min. Sections were incubated with rabbit polyclonal antibody against claudin 1 (catalog no. 51–9000, dilution 1 : 100; Thermo Fischer, Rockford, IL, USA). Deparaffinized sections of the same thickness were pretreated with citrate-buffer at pH 6.0 for 20 min, and incubated with claudin 4 (catalog no. 32–9400, dilution 1 : 100, Thermo Fischer). We used 3,3′-diaminobenzidine tetrahydrochloride (Sigma, St Louis, MO, USA) for specific immunostaining, with final counterstaining with hematoxylin. The total staining process was carried out on a Leica BOND-MAX immunostainer (Leica Biosystems, Newcastle, UK). Skin and colon tissues were used as a positive control, and an additional section from the corresponding block without primary antibody was used as a negative control for claudin 1 and 4. The same two pathologists also reviewed the positive and negative controls.

### 2.3. Evaluation of Immunohistochemistry

We considered cells with membranous and/or cytoplasmic immunoreactivity for both claudin 1 and 4 as positive, and semiquantitatively analyzed each case by determining the percentage of positive cells. Each case was categorized into one of the four groups: 0, negative; 1+, 1–30%; 2+, 31–80%; and 3+, >80%. Increased or decreased expressions of claudins in BCC were determined in comparison with expressions in the corresponding normal skin.

### 2.4. Statistical Analysis

We used *χ*^2^ tests to compare the frequencies at which BCCs had IHC scores of 0 or 1+ versus 2+ or 3+. Values of *P* < 0.05 were considered significant. Data analysis was performed with SPSS version 11.0 for Windows (SPSS, Tokyo, Japan).

### 2.5. Ethics Approval

This study adhered to the tenets of the Declaration of Helsinki. Informed consent was obtained from all patients prior to surgery. Skin tissues were collected in accordance with the protocol approved by local ethics committees at Tokyo Women's Medical University (Shinjuku, Tokyo, Japan; no. 2022-0026).

## 3. Results

**Figure 1:**
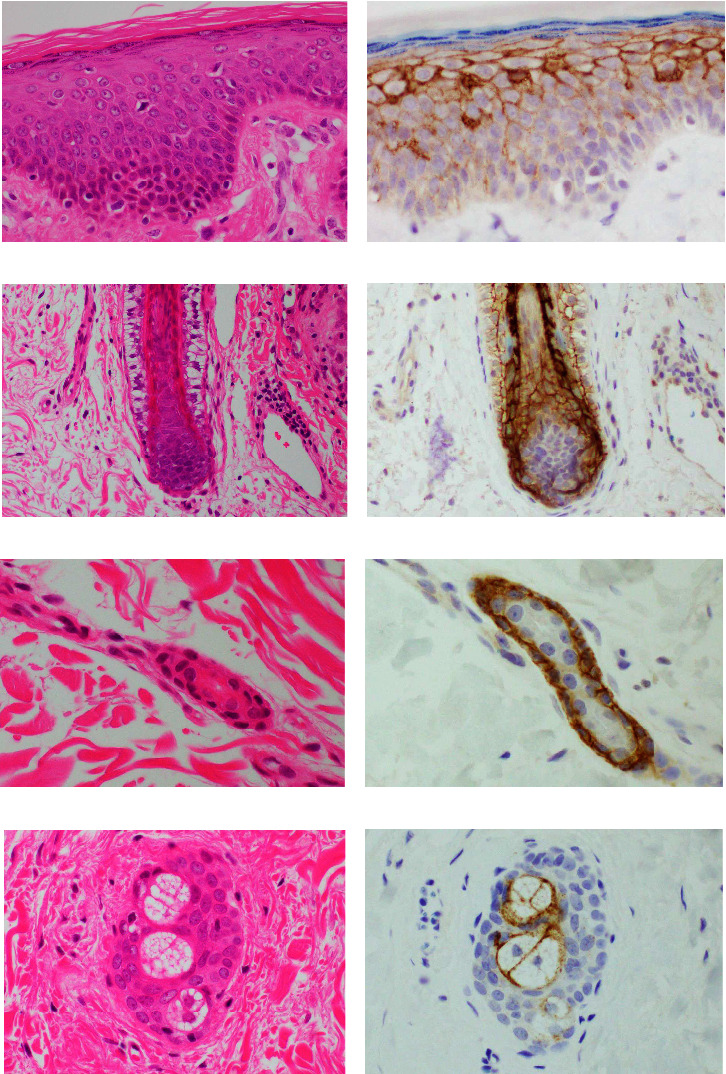
Representative immunohistochemical results for claudin 1 (b, d, f, and h) and corresponding HE-stained sections (a, c, e, and g) in normal skin. (a) Microphotograph of epidermis. (b) Reactivity is observed in the membrane of the basal to granular layer. Staining intensity is strongest in the stratum spinosum with occasional cytoplasmic reactivity. (c) Microphotograph of a hair follicle. (d) Hair follicle is positive and intensity is highest in the membrane and cytoplasm of the inner root sheath. (e) Microphotograph of a sweat gland. (f) Myoepithelial cells are positive for claudin 1. (g) Microphotograph of a sebaceous gland. (h) Membranous reactivity for claudin 1 is seen in sebaceous cells.

Claudin 1 expression in normal skin is shown in Figure 1. Localization of positive cells and intracellular positive sites is summarized in Table 1. The granular to basal layer of epidermis showed reactivity (Figures 1(a) and 1(b)). Immunoreactivity was most intense in the prickle cell layer, in which scattered epithelia showed cytoplasmic reactivity along with the reactivity in the cell membrane. Hair follicles appeared diffusely positive. Membranous and cytoplasmic positivity was observed in the inner root sheath with the strongest staining intensity (Figures 1(c) and 1(d)). Myoepithelial cells of sweat glands showed positivity in the membrane and cytoplasm (Figures 1(e) and 1(f)). Membranous reactivity was observed in sebaceous cells (Figures 1(g) and 1(h)).

**Figure 2:**
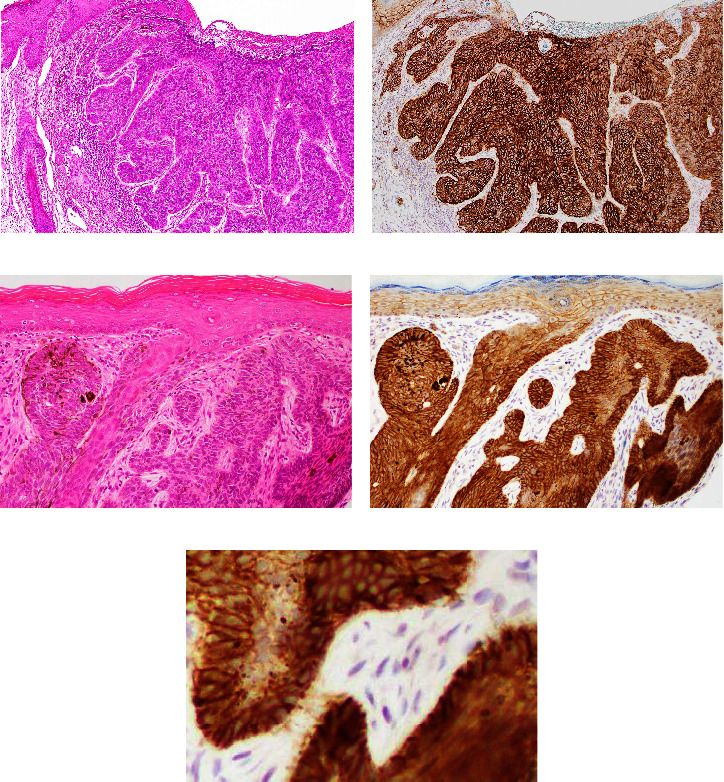
Representative immunohistochemical results for claudin 1 (b, d, and e) and corresponding HE-stained sections (a and d) in lower-grade BCC. (a) Microphotograph of nodular BCC. (b) Neoplastic cells are diffusely and strongly positive in cytoplasm and membrane. (c) Microphotograph of BCC and adjacent epidermis. (d) BCC shows strong membranous and cytoplasmic reactivity in the periphery of tumour nests. Reactivity appears more intense in BCC than in normal epidermis (arrow). Claudin 1 was positive with weaker staining intensity in the central area of tumour nest (arrow head). (e) Positivity is seen in the membrane and cytoplasm.

Representative results for claudin 1 in BCC are shown in Figure 2. The localization of positive BCC cells and positive site is summarized in Table 1. Almost all BCC cells showed high, diffuse expression of claudin 1 (Figures 2(a) and 2(b)). Staining intensity showed intratumoral heterogeneity in some BCCs. Strong membranous and cytoplasmic positivity tended to be observed in neoplastic cells showing nuclear palisading in the periphery of tumour nest (Figures 2(c)–2(e)). Conversely, reactivity attenuated and occasionally disappeared in the central area of tumour nests.

**Figure 3:**
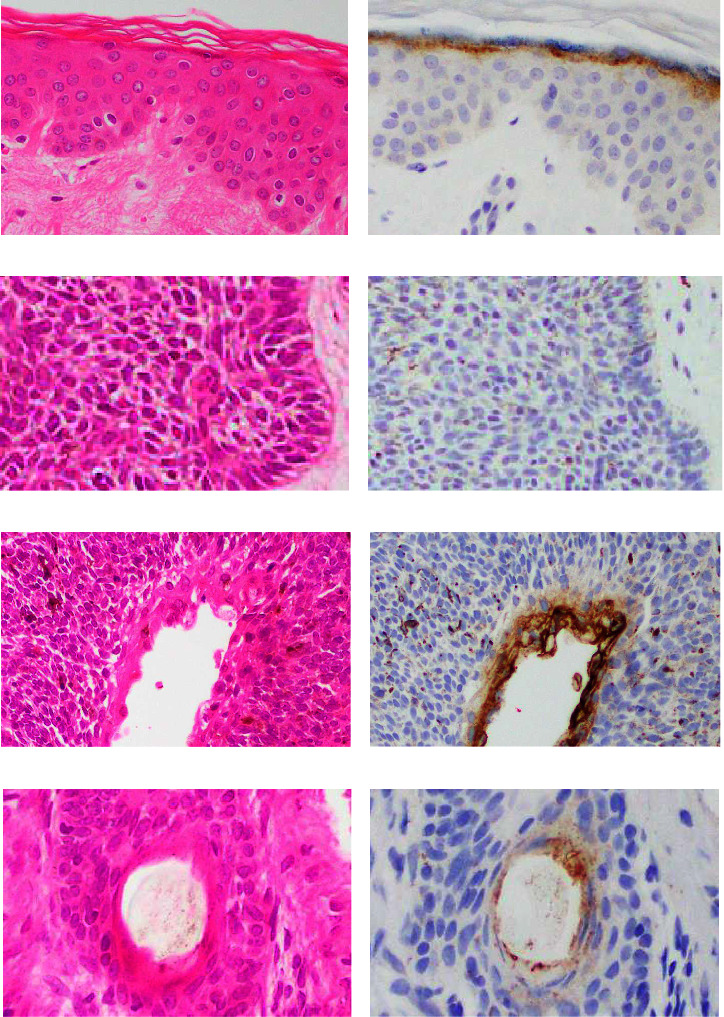
Representative results for claudin 4 (b, d, f, and h) and corresponding HE-stained sections (a, c, e, and g) in normal skin and lower-grade BCC. (a) Microphotograph of normal epidermis. (b) Cytoplasm of epithelia in the granular layer appears positive. (c, e, and g) microphotographs of HE-stained sections in BCC. (c) Neoplastic cells appear uniformly negative. (d) Reactivity is observed in neoplastic cells with differentiation towards the keratinizing layer. (e) Positivity is limited to neoplastic cell showing follicular differentiation (arrow).

IHC results for claudin 4 in normal skin and lower-grade BCC are shown in Figure 3. Localization of positive cell and intracellular distributions is summarized in Table 1. Cytoplasmic expression was detected in the stratum granulosum of non-neoplastic epidermis (Figures 3(a) and 3(b)). Types of claudin 4-positive cells in dermal appendages were congruent with those of claudin 1 (Table 1; figures not shown). BCC mostly lacked immunoreactivity for claudin 4 (Figures 3(c) and 3(d)). Lower-grade BCCs showed focal positivity. Positive cells were limited to neoplastic cells showing keratinizing (Figures 3(e) and 3(f)) and follicular (Figures 3(g) and 3(h)) differentiation.

Clinicopathological features and IHC scores of claudin 1 and 4 in 36 BCCs are summarized in Table 2. Patients ranged in age from 43 to 90 years old (mean age 71.1 years), with 15 men and 21 women. Microscopic examination revealed that BCC variants comprised 34 lower-risk tumours (23 nodular, 8 superficial, 2 infundibulocystic, and 1 fibroepithelial) and two higher-risk tumours (1 micronodular and 1 infiltrating). The follow-up intervals ranged from 1 to 60 months (mean, 12.8 months). All patients remained alive without recurrence from the surgery.

IHC claudin scores of 36 BCCs are shown in Table 2. All BCCs were positive for claudin 1. In 33 of 34 lower-grade BCCs, we detected claudin 1-IHC scores of 2+ and 3+. IHC scores of claudin 1 were higher in all lower-grade BCCs than in adjacent normal skin. Conversely, 13 and 15 of the 34 lower-grade BCCs showed scores of 1+ and 0 for claudin 4. Claudin 4 IHC was lower in all lower-grade BCCs than in normal skins.

Regarding higher-grade BCCs, one infiltrative BCC scored 3+ for claudin 1 and 0 for claudin 4. Claudin 1 was increased and claudin 4 was decreased in this BCC. One micronodular BCC displayed scores 1+ for claudin 1 and 2+ for claudin 4. The corresponding normal tissue displayed scores of 2+ for claudin 1 and 1+ for claudin 4.

Frequencies for IHC scores for claudin 1 and 4 in BCCs are shown in Table 3. Claudin 1-IHC scores of 0, 1+, 2+, and 3+ were detected in 0 (0%), 2 (5.5%), 11 (30.5%), and 23 (64%) of the 36 BCCs, respectively. Frequencies of BCCs with scores of 2+ and 3+ for claudin 1 were significantly higher than those of 0 and 1+ (*P* < 0.05). Conversely, more than half of BCCs (22/36, 61%) were negative for claudin 4 (IHC score 0). Claudin 4-IHC scores gradually decreased, with scores of 1+, 2+, 3+ in 13 (36%), 1 (3%), and 0 (0%). BCCs demonstrated a significantly higher ratio of 0 and 1+ than of 2+ and 3+ for claudin 4-IHC scores (*P* < 0.05).

**Figure 4:**
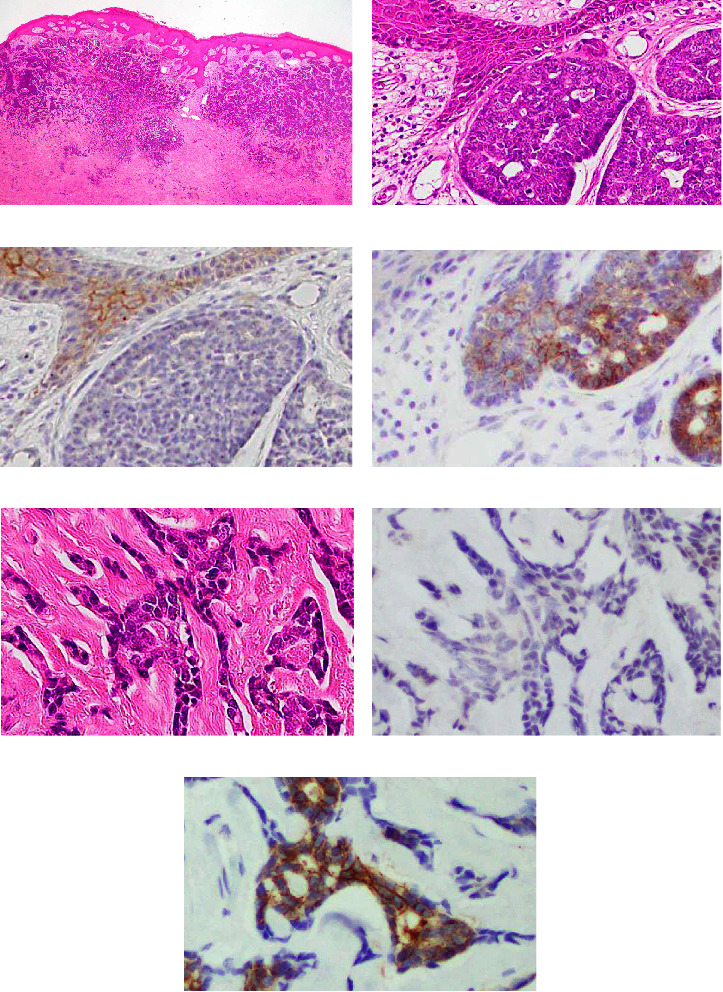
Immunohistochemical results for claudin 1 (c and f) and claudin 4 (d and g) and corresponding HE-stained sections (b and e) of micronodular BCC (case 1). (a) Micronodular pattern is observed in at low magnification in HE-stained section. (b) BCC cells display adenoid cystic architecture in some areas. In these areas, neoplastic cells are negative for claudin 1 (c) and positive for claudin 4 (d). (e) BCC cells show infiltrative growth arranged in a trabecular pattern in deep areas. In these areas, neoplastic cells are also negative for claudin 1 (f) and positive for claudin 4 (g). (h) Reactivity for claudin 4 is observed in the membrane and cytoplasm.

Figure 4 shows microscopic features and IHC results for claudin 1 and 4 in micronodular BCC from Case 1. The BCC displayed a multinodular growth pattern under low-power magnification and was designated as “micronodular variant” (Figure 4(a)). In some areas of the BCC, neoplastic cells were arranged in an adenoid cystic pattern beneath the covering non-neoplastic epidermis (Figure 4(b)). In these areas, the majority of neoplastic cells lacked reactivity for claudin 1 (Figure 4(c)), but was diffusely positive for claudin 4 (Figure 4(d)). BCC cells were arranged in a trabecular pattern at a deep infiltrative area (Figure 4(e)). In this area, neoplastic cells were completely devoid of reactivity for claudin 1 (Figure 4(f)), whereas claudin 4 was strongly expressed in both membrane and cytoplasm (Figure 4(g)).

## 4. Discussion

Claudin family members mainly constitute TJs. Numerous report have provided evidence regarding claudin status in the tumours of various organs. Claudin expression was upregulated in some carcinomas, but downregulated in others [13, 14]. Such a contradictory pattern seems to be determined by the organ from which the carcinoma originates, even though each tumour belongs to the same histological subtype [10]. Furthermore, the expression status of the claudin family depends on the tumour stage. Taken together, claudin expression alone is insufficient to explain the correlations with cancer biology.

A decrease or loss of claudin 1 was strongly associated with poor patient prognosis for colon cancer [15]. Claudin 1 expression was also attenuated in aggressive carcinomas with unfavorable clinical outcomes among prostatic [16] and lung [17] adenocarcinomas. Claudins maintain cell-to-cell adhesion and their loss or decrease leads to a loss of polarity, poor differentiation, and increase invasive capability. Stable membranous claudin expression can be reasonably considered to act as a suppressor of cancer metastasis and dedifferentiation. The present IHC revealed that neoplastic cells in lower-risk BCCs were highly expressive of claudin 1 in the membranes. Claudin 1 may therefore be functionally retained even after carcinogenic processes, resulting in stiff cell-to-cell connections. Furthermore, the high membranous expression of claudin 1 may explain why BCC quite infrequently metastasize to distant organ via lymphovascular channel. Intriguingly, our results showed that one multinodular BCC displayed low expression of claudin 1, indicating a possible correlation with its increased metastatic capability.

Interpreting the significance of upregulated claudin for cancer growth and expansion is relatively different. The increased paracellular permeability of nutrients and growth factors may be beneficial for cancer cell growth and invasiveness via accelerated signal transduction and a favorable microenvironment. Claudin 1 also promotes epithelial-mesenchymal transition (EMT) and cell migration by facilitating tumour necrosis factor (TNF)-*α* [18] or the c-Abl-ERK [19] signaling pathway. For instance, an increase in claudin 3 and 4 is relevant to increases in cell invasion, motility, and survival of ovarian cancer cells [20]. In addition, high claudin 1 expression correlates significantly with a more invasive phenotype of pancreatic intraductal mucinous neoplasms [21]. In this study, claudin 1 appeared to be upregulated in all lower-grade BCCs and claudin 4 was upregulated in one higher-grade BCC. Such claudin increases may contribute to the promotion of cancer cell proliferation and generation of a suitable microenvironment for cancer cells.

Some cancer cells aberrantly express claudin 1 in the cytoplasm. TJ proteins display a dynamic consecutive recycling between the plasma membrane and intracellular vesicle in MDCK cells [22]. The aberrant cytoplasmic expression of claudin can be explained by endocytic trafficking. Claudin 1 expression in cytoplasm along with the membrane was encountered at the invasive front of tongue SCC [23]. In particular, the intracellular expression of claudin 1 correlates significantly with a greater frequency of lymph node metastasis through the promotion of cell mobility. Claudin 1 was also reported to promote invasive activity by disrupting the extracellular matrix via EMT in oral SCC [24]. In our study, BCC cells simultaneously expressed claudins in membranes and cytoplasm. In particular, claudin 1 was strongly expressed in the membranes and cytoplasm of the BCC cells mainly comprising the periphery of tumour nests. Claudin 4 was also upregulated in membranes and cytoplasm of one higher-risk BCC cell. Accordingly, we speculate that upregulated membranous and cytoplasmic expression of claudins promotes local invasiveness by disrupting the characteristic fibromyxoid stroma of BCC via the EMT machinery.

Pathologists occasionally encounter problems in the pathological diagnosis of microscopically mimicking tumours, such as lung adenocarcinoma and malignant mesothelioma. These tumours sometimes share morphological features and are difficult to distinguish from each other merely based on morphology. Jo et al. reported that claudin 4-IHC served as a useful diagnostic marker for lung adenocarcinoma [25]. According to that report, all mesotheliomas were negative for claudin 4, while almost all adenocarcinomas were positive in cytology specimens of pleural effusion. Our results apparently demonstrated high claudin 1 expression and low claudin 4 expression in lower-risk BCCs. Likewise, this specific pattern of claudin may offer a useful diagnostic tool for making a pathological diagnosis.

To date, claudin in skin tumours has been poorly elucidated. Most previous investigations have focused on claudin family expression in cSCC. Morita et al. first reported that claudin 1 was heterogeneously expressed and that claudin 4 was decreased or absent [8]. Moderate-to-high claudin 1 expression was then reported [10]. Conversely, claudin 1 was decreased and claudin 4 was variably expressed [7]. More recently, da Cruz Silva et al. reported that cSCC showed focal expression of claudin 1 and diffuse expression of claudin 4 [9]. In the present study, IHC revealed the converse expression pattern in lower-grade BCCs. These results were unlike most previously reported for cSCC.

Claudin 3 and 4 are known to be receptors for CPE-binding sites [5]. CPE induces cytolysis through membranous permeability by binding to its receptors. Claudin 3- andclaudin 4-expressing prostatic cancer cells have been shown to be strikingly sensitive to CPE-mediated cytolysis [26]. Similar effects were successfully obtained for carcinomas of the breast [27], ovary [28], and pancreas [29]. This raises the possibility that claudin 3 and 4 are potential therapeutic targets for these cancers from a clinical perspective. The present investigations using IHC disclosed that claudin 4 was significantly upregulated in one case of higher-grade BCC. Claudin 4 may therefore be a candidate as therapeutic target for uncontrollable claudin 4-positive BCC. Nevertheless, tumour-specific drug delivery will be required since claudin 3 and 4 are highly expressed in normal gut, lung, and kidney, all of which are indispensable for survival. Furthermore, pharmacological test remains necessary to resolve this problem.

## 5. Conclusion

Our results indicated that most lower-risk BCCs are highly expressive of claudin 1 and poorly expressive of claudin 4. This expression pattern may serve as a useful ancillary tool for making pathological diagnosis of lower-risk BCC. Of note, one of the two higher-risk BCCs showed the converse pattern of expressions. This may be the characteristic of higher-risk BCC, but further analysis in a large series is necessary. A membranous localization of claudin 1 was evident when comparing in lower-grade BCCs with the normal skin. The abundant membranous claudin may explain the behavioral property of BCC showing markedly infrequent metastasis. It also raises the possibility that the cytoplasmic expressions of claudins reinforce the local invasiveness of BCC with fibromyxoid stroma via EMT and signal transduction. In the future, increased membranous and cytoplasmic claudins may be expected to provide therapeutic targets for uncontrollable BCC.

## Figures and Tables

**Table 1:** Localization of claudin 1- and claudin 4-positive cells in normal skin and basal cell carcinoma.

*Normal skin*		*Basal cell carcinoma*	
Location	Intracellular distribution	Location	Intracellular distribution
*Claudin 1*			
Epidermis, basal to granular cell layer (staining intensity is strongest in pickle cell layer)	Membrane^※^	Majority of neoplastic cells (staining intensity is attenuated in the central area of tumour nests)	Membrane and cytoplasm
Myoepithelial cell of sweat gland	Membrane and cytoplasm		
Sebaceous cell of sebaceous gland	Membrane		
Hair follicle	Membrane^※^		

*Claudin 4*			
Epidermis, granular layer	Cytoplasm	Neoplastic cells showing glandular or adenoid cystic structure, sebaceous or keratinizing in the central area of tumour nests	Membrane and cytoplasm
Myoepithelial cell of sweat gland	Membrane and cytoplasm		
Sebaceous cell of sebaceous gland	Membrane		
Hair follicle	Membrane and cytoplasm		

^※^inner root sheath of hair follicle and small population of pickle layer showed positivity in cytoplasm along with membrane.

**Table 2:** Clinicopathological features and IHC scores of claudin 1 and 4 in basal cell carcinoma.

Case nos.	Age	Gender	Histological sybtype	*Immunohistochemical score*		Prognosis (follow-up, month)
				Claudin 1	Claudin 4	
1	90	M	Micronodular	1+	2+	AWOR (8)
2	76	M	Infiltrating	3+	0	AWOR (13)
3	88	F	Nodular	3+	1+	AWOR (18)
4	69	F	Nodular	3+	1+	AWOR (10)
5	80	M	Nodular	3+	1+	AWOR (5)
6	73	F	Nodular	3+	1+	AWOR (60)
7	61	M	Nodular	3+	1+	AWOR (2)
8	71	F	Nodular	3+	0	AWOR (12)
9	78	M	Nodular	3+	0	AWOR (12)
10	81	F	Nodular	3+	0	AWOR (2)
11	57	F	Nodular	3+	0	AWOR (14)
12	85	F	Nodular	3+	0	AWOR (10)
13	57	M	Nodular	3+	0	AWOR (13)
14	60	M	Nodular	3+	0	AWOR (20)
15	77	F	Nodular	3+	0	AWOR (21)
16	89	M	Nodular	3+	0	AWOR (6)
17	74	F	Nodular	3+	0	AWOR (43)
18	82	M	Nodular	2+	1+	AWOR (6)
19	84	F	Nodular	2+	1+	AWOR (18)
20	46	M	Nodular	2+	1+	AWOR (2)
21	79	M	Nodular	2+	1+	AWOR (11)
22	82	M	Nodular	2+	0	AWOR (12)
23	48	M	Nodular	2+	0	AWOR (1)
24	79	F	Nodular	2+	0	AWOR (16)
25	70	F	Nodular	1+	0	AWOR (15)
28	80	M	Superficial	3+	1+	AWOR (12)
26	75	F	Superficial	3+	0	AWOR (6)
27	77	F	Superficial	3+	0	AWOR (6)
30	54	F	Superficial	3+	0	AWOR (20)
32	51	F	Superficial	3+	0	AWOR (8)
33	87	F	Superficial	3+	0	AWOR (22)
29	74	F	Superficial	2+	0	AWOR (3)
31	74	F	Superficial	2+	0	AWOR (4)
34	63	M	Infundibulocystic	2+	1+	AWOR (1)
35	43	F	Infundibulocystic	2+	1+	AWOR (17)
36	47	F	Fibroepithelial	3+	1+	AWOR (12)

F: female; M: male; AWOR: alive without recurrence.

**Table 3:** Frequencies of claudin IHC scores in 36 basal cell carcinomas.

IHC scores	0	1+	2+	3+
Claudin 1	0 (0%)	2 (5.5%)	11 (30.5%)^*∗*^	23 (64%)^*∗*^
Claudin 4	22 (61%)	13 (36%)	1 (3%)^†^	0 (0%)^†^

^*∗*^ *P* < 0.05 (score 0 and 1+ versus score 2+ and 3+ of claudin 1). ^†^*P* < 0.05 (score 0 and 1+ versus score 2+ and 3+ of claudin 4).

## Data Availability

The data used to support the findings of this study are included within the article.
